# Deep brain stimulation for patients with refractory epilepsy: nuclei selection and surgical outcome

**DOI:** 10.3389/fneur.2023.1169105

**Published:** 2023-05-12

**Authors:** Hao Yan, Xueyuan Wang, Xiaohua Zhang, Liang Qiao, Runshi Gao, Duanyu Ni, Wei Shu, Cuiping Xu, Liankun Ren, Tao Yu

**Affiliations:** ^1^Department of Functional Neurosurgery, Beijing Institute of Functional Neurosurgery, Xuanwu Hospital, Capital Medical University, Beijing, China; ^2^Department of Neurology, Comprehensive Epilepsy Center of Beijing, Beijing Key Laboratory of Neuromodulation, Xuanwu Hospital, Capital Medical University, Beijing, China

**Keywords:** deep brain stimulation, refractory epilepsy, anterior nucleus of the thalamus, subthalamic nucleus, centromedian nucleus

## Abstract

**Objective:**

By studying the surgical outcome of deep brain stimulation (DBS) of different target nuclei for patients with refractory epilepsy, we aimed to explore a clinically feasible target nucleus selection strategy.

**Methods:**

We selected patients with refractory epilepsy who were not eligible for resective surgery. For each patient, we performed DBS on a thalamic nucleus [anterior nucleus of the thalamus (ANT), subthalamic nucleus (STN), centromedian nucleus (CMN), or pulvinar nucleus (PN)] selected based on the location of the patient's epileptogenic zone (EZ) and the possible epileptic network involved. We monitored the clinical outcomes for at least 12 months and analyzed the clinical characteristics and seizure frequency changes to assess the postoperative efficacy of DBS on the different target nuclei.

**Results:**

Out of the 65 included patients, 46 (70.8%) responded to DBS. Among the 65 patients, 45 underwent ANT-DBS, 29 (64.4%) responded to the treatment, and four (8.9%) of them reported being seizure-free for at least 1 year. Among the patients with temporal lobe epilepsy (TLE, *n* = 36) and extratemporal lobe epilepsy (ETLE, *n* = 9), 22 (61.1%) and 7 (77.8%) responded to the treatment, respectively. Among the 45 patients who underwent ANT-DBS, 28 (62%) had focal to bilateral tonic-clonic seizures (FBTCS). Of these 28 patients, 18 (64%) responded to the treatment. Out of the 65 included patients, 16 had EZ related to the sensorimotor cortex and underwent STN-DBS. Among them, 13 (81.3%) responded to the treatment, and two (12.5%) were seizure-free for at least 6 months. Three patients had Lennox–Gastaut syndrome (LGS)-like epilepsy and underwent CMN-DBS; all of them responded to the treatment (seizure frequency reductions: 51.6%, 79.6%, and 79.5%). Finally, one patient with bilateral occipital lobe epilepsy underwent PN-DBS, reducing the seizure frequency by 69.7%.

**Significance:**

ANT-DBS is effective for patients with TLE or ETLE. In addition, ANT-DBS is effective for patients with FBTCS. STN-DBS might be an optimal treatment for patients with motor seizures, especially when the EZ overlaps the sensorimotor cortex. CMN and PN may be considered modulating targets for patients with LGS-like epilepsy or occipital lobe epilepsy, respectively.

## 1. Introduction

Epilepsy is a chronic neurological disorder that affects approximately 1% of the global population (1, 2). Currently, most patients can benefit from drug therapy, the first-line treatment for epilepsy. However, nearly 30% of patients suffer from drug-resistant epilepsy (3). In these patients, identifying the epileptogenic foci and performing resective surgery may help reduce or even control seizures completely (4). Noteworthily, epilepsy surgery remains challenging, including the difficulty of localizing the seizure focus, multiple seizure foci, and seizure focus close to the eloquent cortex (5, 6). Accordingly, not all patients with drug-resistant epilepsy may benefit from surgical resection. Therefore, alternative options are urgently needed (7).

Neurostimulation is an alternative treatment for patients who reap limited benefits from resective surgery (8). In the 1970s and 1980s, deep brain stimulation (DBS) emerged as an approach for treating epilepsy by stimulating a specific target (9, 10). Although the specific antiepileptic mechanism remains to be detailed, numerous clinical reports have confirmed the effectiveness of DBS against epilepsy. Gastaut and Broughton proposed that focal epilepsy is a cortico-subcortical disorder and suggested that subcortical structures participate in seizure initiation (11). Previous studies had documented that the thalamus had a widespread interactive connection with cortical regions and might, as a critical subcortical structure, participate in all focal epilepsies independently of the etiology or focus localization (12). Therefore, it seems reasonable to consider the thalamus as the stimulation target.

The famous SANTE (Stimulation of the Anterior Nucleus of the Thalamus for Epilepsy) clinical study has demonstrated the safety and effectiveness of the anterior nucleus of the thalamus (ANT)-DBS (13). Subsequent studies confirmed the efficacy of ANT-DBS (14, 15). However, whether all patients would benefit from ANT-DBS remains a crucial clinical question. In other words, is the ANT the best modulating target for patients with different epilepsy or seizure types? In our opinion, due to the complexity of the thalamus anatomy and the functional network, one of the challenges of the DBS treatment for epilepsy is choosing the optimal stimulation target for specific epilepsy or different seizure types. According to limited clinical studies, DBS can also be effective on other nuclei, such as the subthalamic nucleus (STN) (16), the centromedian nucleus (CMN) (17), and the pulvinar nucleus (PN) (18). The present single-center study reports the effect of DBS on different thalamic nuclei for drug-resistant epilepsy. It provides new insights for selecting the optimal nuclei target for patients with refractory epilepsy.

## 2. Methods

### 2.1. Patient selection

All the participants were diagnosed with drug-resistant epilepsy at the Beijing Institute of Functional Neurosurgery, Xuanwu Hospital, between January 2012 and December 2021. In total, this study included 65 patients. Their mean age was 24.37 ± 9.56 years and 36.9% (24 subjects) were women. The mean duration of epilepsy was 12.76 ± 7.28 years. The mean follow-up duration was 39.4 ± 20.9 months (ranging from 12 to 108 months). Experienced neurologists and neurosurgeons discussed and designed the selection of the stimulation target and the surgical plan of the DBS for each patient.

The inclusion criteria were as follows: (1) Patients diagnosed with drug-refractory epilepsy were based on the ILAE Classification of Epilepsies (19). (2) The preoperative evaluation indicated that the patient was inoperable or had contraindications for resective surgery, such as widely distributed epileptogenic zones (EZ), EZ located in the functional cortex, failed resective surgery, or the patient refused to undergo the resective surgery.

### 2.2. Presurgical evaluation

We performed long-term scalp video electroencephalography (VEEG) to record at least three habitual seizures for each patient using a video EEG monitoring system (Micromed, Treviso, Italy). In some patients, we identified the EZ by performing stereotactic electroencephalography (SEEG). All patients underwent a high-resolution magnetic resonance imaging (MRI) protocol performed using a 3.0-T MR Scanner (Siemens, Verio, Germany) and consisting of conventional axial, sagittal, and coronal T1-weighted spin-echo sequences. In some patients, we identified the EZ by performing magnetoencephalography and positron emission tomography-computed tomography. The patients who underwent the DBS procedure after the special committee consultation excluded resective surgery based on their clinical data. For each patient, we selected the target thalamic nucleus for DBS (ANT, STN, CMN, or PN) based on the patient's epilepsy or seizure type and the location of the epileptogenic focus, as well as the possible epileptic network involved (20, 21). We defined the baseline for each patient as their mean seizure frequency over the 3-month pre-implant period.

### 2.3. Surgical method

We implanted the DBS electrodes (Model 3387 or 3389; Medtronic, Inc., Minneapolis, MN, USA) with the assistance of a frame-based, microelectrode-guided, stereotactic technique under general anesthesia. All the patients receiving ANT-DBS, CMN-DBS, and PN-DBS underwent bilateral electrode implantation. In patients with STN-DBS, some patients with specific epilepsy types (such as those with the possible EZ located in the unilateral hemisphere) underwent unilateral electrode implantation. With the help of the high-resolution T1-weighted images, we delineated the thalamus nuclei based on the Morel Stereotactic Atlas. We performed the surgical procedure of the implantation of the DBS leads and the pulse generator (Model 3628 screener, Medtronic, Inc., Minneapolis, MN, USA) based on previous studies (22). Postoperative computed tomography was performed and registered with the T1-weighted images to confirm the locations of the electrodes.

### 2.4. Postoperative follow-up and parameters adjustment

One month after the implantation procedure, the pulse generator was initiated to be activated and programmed. The outpatient review of each patient was carried out 3 months after the operation to identify the occurrence of long-term complications. In addition, the stimulation parameters and contacts were adjusted based on seizure frequency and clinical response. We categorized patients with a ≥50% decrease in seizure frequency (mean seizure frequency for the last 3 months of follow-up, compared with the baseline as responders and patients with a < 50% decrease in seizure frequency as non-responders. All of the patients were followed up monthly or trimonthly, and the seizure frequency was reported by the patient or the family members. Noteworthy, the post-operative seizure needs to be verified with the habitual seizure. The data were recorded from outpatient reviews, medical record reviews, patients' daily diaries, and telephone interviews. The postoperative program control details for each patient were also documented.

## 3. Results

**Figure 1:**
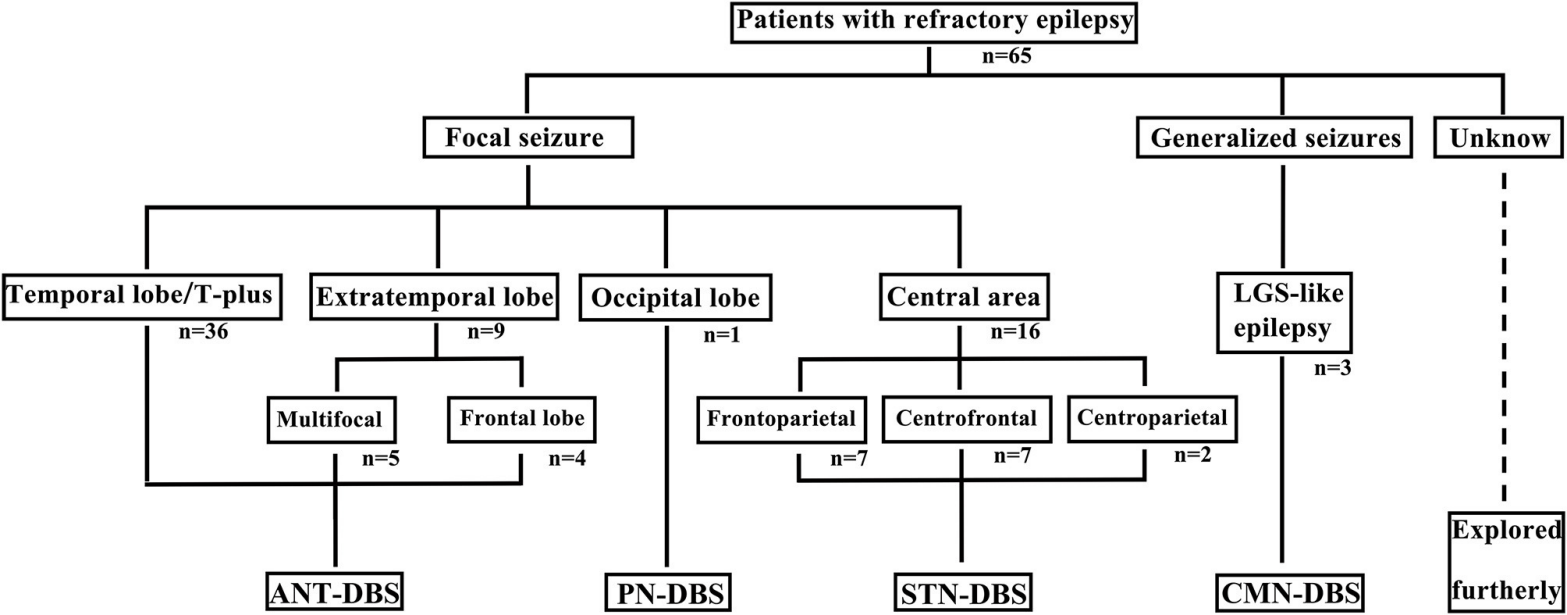
Different DBS procedures were selected for patients with various epilepsy or seizure types. T, temporal; LGS, Lennox–Gastaut syndrome.

**Figure 2:**
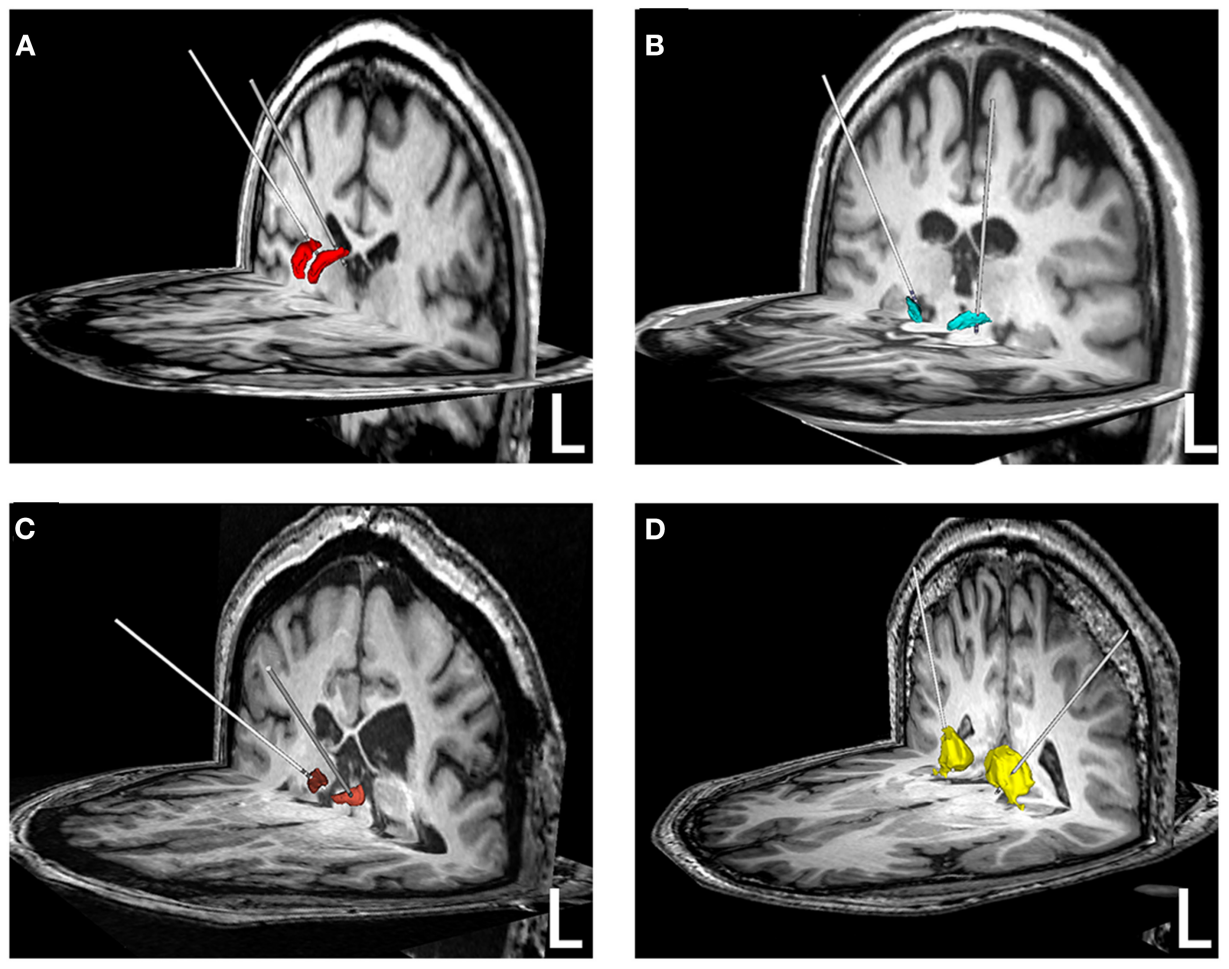
Reconstruction of electrodes in different brain nuclei. **(A)** ANT-DBS; **(B)** STN-DBS; **(C)** CMN-DBS; **(D)** PN-DBS. The ANT (red), STN (green), CMN (brown), and PN (yellow) were reconstructed based on the Morel Stereotactic Atlas.

Among the 65 patients, 45 underwent ANT-DBS, 16 underwent STN-DBS, three underwent CMN-DBS, and one underwent PN-DBS (Figures 1, 2). The demographic data and clinical characteristics of these patients are presented in Table 1 and Supplementary Table 1.

**Table 1:** Characteristics of the included patients.

**Characteristics of patients (*n =* 65)**	**ANT-DBS (*n =* 45)**	**STN-DBS (*n =* 16)**	**CMN-DBS (*n =* 3)**	**PN-DBS (*n =* 1)**
Age (y)	29.7 ± 9.59	19.56 ± 7.99	16.00 ± 4.00	23
Duration of epilepsy (y)	12.87 ± 7.80	12.29 ± 6.45	11.33 ± 4.04	20
Females^#^	15 (33%)	6 (37.5%)	3 (100%)	0
Mean follow-up (m)	44.24 ± 17.55	26.63 ± 23.02	43.6 ± 32.01	13
**Seizure characteristic** ^*^				
Focal seizure	45 (69.2%)	16 (24.6%)	3 (4.6%)	1 (1.5%)
Focal to bilateral tonic-clonic seizures	28 (43.1%)	7 (10.8%)	3 (4.6%)	1 (1.5%)
Motor seizures	0	16 (24.6%)	0	0
**Location of EZ** ^#^				
T/ T-Plus	36 (80%)	0	0	0
F-C/ C-P	4 (9%)	16 (100%)	0	0
O	0	0	0	1 (100%)
Multifocal	5 (11%)	0	Unknown	0
**Surgical outcome** ^#^				
Responders	29 (64.4%)	13 (81.3%)	3 (100%)	1 (100%)
Seizure frequency reduction	37 (82.2%)	14 (87.5%)	3 (100%)	1 (100%)

* The proportion of individuals in total patients.

# The proportion of individuals in patients with specific DBS procedure.

T, temporal; T-Plus, temporal plus; F, frontal; C, central area; P, parietal; O, occipital; FBTCS, focal to bilateral tonic-clonic seizures; EZ, epileptogenic zone; y, year; m, month.

In our study, 46 of the 65 patients (70.8%) were responders (average decrease in seizure frequency 81.2%, ranging from 51.6% to 100%, interquartile range [IQR] 33.75). Among the 19 non-responders, nine patients experienced varying degrees of decrease in seizure frequency but <50% compared with the baseline (average 26.9%, range 9.1%−47.8%, IQR 25.75). Six patients reported no significant changes in seizure frequency. In total, four patients reported that their seizure frequency increased to various degrees (ranging from −25% to −220%).

Among the 45 patients who underwent ANT-DBS, 29 (64.4%) were responders (average decrease in seizure frequency 79.7%, ranging from 52.8% to 100%, IQR 31.99) and four (8.9%, patients 1, 5, 26, and 35) reported being seizure-free for at least 1 year. Based on the EEG, symptomatology, and other presurgical data, 36 of the 45 patients (80%) were diagnosed with temporal lobe epilepsy (TLE) and 22 of them (61%) were responders (average 80.2%, range 53.3%−100%, IQR 31.10). Among patients with TLE, nine (20%) were diagnosed with temporal plus (T-plus) epilepsy and eight of them (89%) were responders (average 84.8%, range 61.7%−100%, IQR 33.30). Based on the MRI images, 14 of the 36 patients (39%) with TLE had bilateral hippocampal sclerosis (HS) and eight of them (57.1%) were non-responders (ranging from −220% to 15%). Out of the 45 ANT-DBS patients, nine (20%) were diagnosed with extratemporal lobe epilepsy (ETLE), and seven of them (78%) were responders (average 78.2%, ranging from 52.8% to 100%, IQR 40.00). Among patients with ETLE, four were diagnosed with frontal lobe epilepsy, and three of them were responders (average 86.7%, ranging from 60.0% to 100%); five patients were diagnosed with multifocal epilepsy, and four of them were responders (average 71.9%, ranging from 52.8% to 91.7%, IQR 29.64). All of the patients' seizure types were focal seizures, and 28 of the 45 ANT-DBS patients (62%) had focal to bilateral tonic-clonic seizures (FBTCS). Among the 28 patients with FBTCS, 18 (64%) were responders (average 78.4%, ranging from 52.8% to 100%, IQR 31.88). Noteworthily, two non-responders with FBTCS reported that their seizure frequency was not significantly reduced, while their seizure severity was significantly improved (i.e., the duration of the seizures was reduced, and the patients quickly regained consciousness after the seizures).

In patients who underwent STN-DBS (*n* = 16), the etiologies were diverse, with cases of schizencephaly (*n* = 5), focal cortical dysplasia (*n* = 5), gray matter heterotopia (*n* = 3), and encephalitis (*n* = 3). One patient had both schizencephaly and focal cortical dysplasia. Noteworthily, the EZ of all patients was associated with the sensorimotor cortex, namely, with its centrofrontal (*n* = 7), centroparietal (*n* = 2), and frontoparietal (*n* = 7) lobes. Among the 16 patients, nine (56%) had motor seizures, while the seven others (44%) presented focal motor seizures and FBTCS. Moreover, 13 of the 16 patients (81%) were responders (average 87.1% reduction, ranging from 54.2% to 100%, IQR 27.58), and two patients (13%, patients 55 and 60) remained seizure-free for at least 6 months. Among the three non-responders, patient 50 suffered motor seizures and FBTCS; the aware motor seizures disappeared and the FBTCS increased after receiving the STN-DBS procedure, patient 57 reported a 25% increase in seizure frequency, and patient 61 reported a 43% decrease in seizure frequency.

For patients who underwent CMN-DBS (*n* = 3), the EZ was difficult to localize and, based on the EEG abnormalities and symptomatology, they were diagnosed with Lennox–Gastaut syndrome (LGS)-like epilepsy. The patients reported a 51.6%, 79.6%, and 79.5% reduction in seizure frequency at 76, 43, and 12 months of follow-up, respectively. Based on the presurgical evaluation, one patient was diagnosed with bilateral occipital lobe epilepsy, and the possible EZ was difficult to be removed surgically. Finally, the patient underwent PN-DBS. After the PN-DBS with elaborate postoperative program control, his seizure frequency was reduced by 69.7% at 13 months of follow-up.

## 4. Discussion

Deep brain stimulation is an emerging and promising treatment for epilepsy. The effectiveness of DBS is mainly related to the appropriate candidates, the optimal stimulation target, and the elaborate postoperative program control strategy. Currently, there are no specific stimulation target selection criteria for the treatment of epilepsy using DBS on the thalamus. Based on the symptomatology, VEEG/SEEG recordings, and imaging information, we inferred epilepsy or seizure type and EZ location for each patient, as well as the possible epileptic network involved. Next, we carefully selected a personalized stimulation target for each patient. We hope that documenting the surgical outcome of DBS in different thalamus nuclei will help clinical decision-makers select the optimal stimulation target for patients with refractory epilepsy.

### 4.1. ANT-DBS

The ANT is the most common stimulation target of DBS in epilepsy treatment (23). The unique anatomical relationship and the functional connection between ANT and the limbic system make this nucleus an ideal stimulation target for TLE treatment (24, 25). In our study, 80% of patients with ANT-DBS had TLE (including T-plus), and our results are in line with previous studies (13). Our previous SEEG study demonstrated that the ANT-DBS would desynchronize the epileptic network in patients with TLE. In addition, the position-specific correlation had also been reported between the DBS applied to the ANT and patients with TLE and EZ within the Papaz circuit or limbic system (26). Moreover, the ANT can receive the interictal period discharges that propagate from the epileptogenic zones in neocortical temporal and mesial temporal epilepsy (27). Therefore, combined with the previous clinical studies, our data suggest that ANT is an optimal stimulation target for patients with TLE. Fasano et al. suggested that patients with frontal seizures also benefit from ANT-DBS (28). The long-term follow-up of the SANTE trial showed that frontal onset seizures also respond well to ANT-DBS (14). In our study, patients with frontal epilepsy showed a good response to ANT-DBS. In addition, patients with multifocal epilepsy also benefited from the ANT-DBS. Previous studies suggested that ANT plays a role in a wider cortical network (29, 30), and other epilepsy types could be treated through ANT stimulation.

In patients with FBTCS, ANT-DBS showed good efficacy, which may be because ANT-DBS modulates the epileptic network excitability. Previous studies suggested that the ANT participates in the organization and maintenance of seizure activity (21). In addition, Tyvaert et al. observed a synchronous activity between the ANT and generalized epileptogenic network in patients with generalized epilepsy, indicating that the ANT is a potential propagation point (31). We speculate that, on the one hand, ANT-DBS might reduce the epileptic network excitability to some extent and raise the seizure threshold, making the seizure less likely to occur. On the other hand, the reduced network excitability might limit the propagation of the epileptic excitatory signal. This hypothesis is also supported by the reduced severity of postoperative seizures in patients with FBTCS. Therefore, based on the mechanism studies and the clinical results, ANT-DBS is also an alternative treatment for patients with FBTCS.

The reasons why some patients have poor responses to ANT-DBS are complex. In our study, ANT-DBS turned out to be poorly effective for patients with bilateral HS. We speculate that the EZ of these patients has excessive excitability, and ANT-DBS may have a relatively weak inhibitory effect on the sclerotic hippocampus. A previous epilepsy study reported that hippocampal DBS was less effective in patients with HS than in patients with normal MRI profiles (32). According to previous studies, the sclerotic hippocampus was related to neuronal reduction, which may prevent the hippocampus to provide enough available tissue for modulation (33). In addition, the sclerotic hippocampus might have an increased impedance and require a more intense stimulus (32). Regarding ANT-DBS in patients with bilateral HS, indirect stimulation based on the specific network may further weaken the regulation effect on the sclerotic hippocampus. Noteworthy, the aberrant circuits may be involved in patients with bilateral HS, which would induce inefficacy or even the paradoxical effect when the ANT-DBS was applied.

Currently, identifying patients who would benefit from ANT-DBS is difficult. Our results indicate that some epilepsy types would be refractory to this treatment. Therefore, different stimulation targets and corresponding surgical indications need to be explored further for patients with refractory epilepsy.

### 4.2. STN-DBS

In some patients with drug-resistant epilepsy, resective surgery would be contraindicated due to the EZ being located in the primary motor cortex. Responsive brain stimulation (34) and ANT-DBS (13) offer an alternative treatment for these patients, but the response is not always satisfactory. In 2002, Benabid et al. first applied STN-DBS to treat epilepsy in a patient with focal centroparietal dysplasia and reported an 80.7% reduction in seizure frequency (16). Subsequent studies confirmed the safety and effectiveness of STN-DBS in patients with motor seizures (35, 36). Regarding the mechanism, our team's prior study demonstrated the interaction between STN and the motor cortex. In addition, STN-DBS with high-frequency stimulation suppressed the interictal spikes and high-frequency oscillations in patients with motor seizures (37).

Based on existing clinical evidence and our knowledge of the mechanism, we choose STN as the target for patients with an EZ overlapping the sensorimotor cortex. STN-DBS significantly reduced motor seizures in these patients in concordance with previous studies (38). Therefore, STN-DBS can be a potent treatment option for patients with motor seizures. Nevertheless, STN-DBS needs to be further investigated in large-scale randomized controlled trials and specific regulative mechanism studies. Notably, patient 57 reported a seizure frequency increase (four times per month) after the STN-DBS, which might be related to the lower baseline (2–3 times per month) and require further stimulation parameter adjustment and follow-up. The EZ of patient 50 was located in the frontoparietal region with focal motor seizures and FBTCS. After STN-DBS, his focal seizures disappeared, and the FBTCS frequency decreased non-significantly; the pulse generator was removed after 14 months due to an increase in FBTCS frequency. We speculated that the poor response may be due to improper stimulation parameters and contacts. Therefore, even when selecting the optimal stimulation target, elaborate postoperative program control is particularly important.

### 4.3. CMN-DBS and PN-DBS

Patients with LGS present specific EEG abnormalities and multiple seizure types, such as generalized tonic seizures (17). Previous studies recorded epileptiform EEG activity in the CMN of patients with generalized tonic seizures from LGS (39). In addition, CMN has diffuse connections with the diffuse frontal areas, brainstem, and striatum, which prompted us to choose the CMN rather than the ANT, as the stimulation target in LGS or LGS-like epilepsy cases (40). Velasco et al. performed CMN-DBS on five patients with drug-resistant epilepsy and reported a significant reduction in secondary generalized tonic-clonic seizures (GTCS) frequency (41). Subsequent randomized controlled and small open-label studies reported significant efficacy for CMN-DBS in generalized seizures, especially in patients with primary or secondary LGS (17, 42). Based on this encouraging clinical data, we performed CMN-DBS in three patients with LGS-like epilepsy and also observed a good response. Therefore, we consider CMN-DBS to be an alternative treatment for patients with generalized-onset epilepsy.

As the largest thalamus nucleus, the PN has extensive connections with areas of the cortex, such as the mesial temporal lobe, the parietal cortex, and the occipital lobe (43–45). The anatomical features of the PN indicate that it is a potential neuromodulation target to treat epilepsy. Compared with the other targets, clinical reports on the stimulation of PN for the treatment of epilepsy are relatively rare. Filipescu et al. investigated PN stimulation on temporal lobe seizures and first suggested that PN-DBS could be a well-tolerated and effective approach for drug-resistant epilepsy (44). In a study of responsive neurostimulation targeting the PN to treat epilepsy, it was effective for drug-resistant epilepsy with posterior quadrant origin (18). In our study, we performed PN-DBS on one patient with bilateral occipital lobe epilepsy, significantly reducing seizure frequency. Although this is only one case, PN does seem to be an alternative target for neuromodulation to treat occipital lobe epilepsy.

### 4.4. Conclusion

Based on our single central clinical results, we summarized empirical guidance for the selection of stimulation targets for patients with refractory epilepsy. Our results show that ANT-DBS is effective for patients with either TLE (including T-plus) or ETLE (including FLE and multifocal epilepsy). However, in patients with bilateral HS, ANT-DBS should be applied with more caution. In addition, ANT-DBS is effective for patients with FBTCS. For patients with motor seizures, especially with the EZ overlapping the sensorimotor cortex, STN-DBS might be a powerful treatment. CMN-DBS and PN-DBS might be alternative options for patients with LGS-like epilepsy and occipital lobe epilepsy, respectively.

### 4.5. Limitation

The small cohort of our study prevented the investigation of the efficacy of DBS in a wider variety of epilepsy and seizure types. In addition, we only reviewed the efficacy of DBS in different thalamus nuclei. In future studies, we would investigate the details of postoperative program control, such as the parameter settings and side effects and the influence factors of surgical outcome.

## Data availability statement

The original contributions presented in the study are included in the article/Supplementary material, further inquiries can be directed to the corresponding authors.

## Ethics statement

The studies involving human participants were reviewed and approved by the Ethics Committee of the Capital Medical University. Written informed consent to participate in this study was provided by the participants' legal guardian/next of kin.

## Author contributions

HY, XW, TY, and LR designed the study. WS, CX, and RG contributed to the analysis of data. XZ, DN, and LQ contributed to the data acquisition. All authors contributed to the manuscript revision and read and approved the submitted version.
