# Supplementary material for: Deep brain stimulation for patients with refractory epilepsy: nuclei selection and surgical outcome
Source: Front Neurol. 2023 May 12;14:1169105. doi: 10.3389/fneur.2023.1169105 (PMC10213517; doi:10.3389/fneur.2023.1169105)
Supplement: Supplementary file 1 [file Table_1.DOCX]

**S-table 1. Clinical data of patients with DBS**

| Patients | Age/ (y) | Epilepsy duration/(y) | Etiology | Seizure type | Location of the EZ | epilepsy type | Site of insertion | Baseline /Seizure frequency before DBS | Seizure frequency at last 3 months follow-up | Seizure reduction | Follow up (m) |
| --- | --- | --- | --- | --- | --- | --- | --- | --- | --- | --- | --- |
| 1 | 13 | 13 | unknown | Focal tonic seizure to bilateral tonic-clonic seizure | F | ETLE | Bi-ANT | 60 | 0 | 100.0% | 65 |
| 2 | 18 | 15 | unknown | Focal tonic seizure to bilateral tonic-clonic seizure | P, operculum | Multifocal | Bi-ANT | 193 | 57 | 70.7% | 77 |
| 3 | 12 | 7 | neonatal intracerebral hemorrhage | Focal tonic seizure to bilateral tonic-clonic seizure | Bi-posterior P | Multifocal | Bi-ANT | 73 | 20 | 72.6% | 53 |
| 4 | 28 | 16 | unknown | Focal impaired awareness tonic seizure | Bi-T | TLE | Bi-ANT | 685 | 88 | 87.2% | 37 |
| 5 | 50 | 36 | meningitis | Focal emotional seizure to bilateral tonic-clonic seizure | T-PLUS | TLE | Bi-ANT | 60 | 0 | 100.0% | 45 |
| 6 | 52 | 36 | unknown | Focal impaired awareness automatisms seizure | Bi-H | TLE | Bi-ANT | 2 | 2 | 0.0% | 50 |
| 7 | 49 | 25 | unknown | Focal automatisms seizure to bilateral tonic-clonic seizure | Bi-T | TLE | Bi-ANT | 2 | 2 | 0.0% | 36 |
| 8 | 29 | 4 | encephalitis | Focal cognitive seizure to bilateral tonic-clonic seizure | F | ETLE | Bi-ANT | 4 | 4 | 15.4% | 37 |
| 9 | 27 | 17 | encephalitis | Focal behavior arrest seizure to bilateral tonic-clonic seizure | Bi-T | TLE | Bi-ANT | 8 | 4 | 47.8% | 68 |
| 10 | 22 | 10 | unknown | Focal tonic seizure to bilateral tonic-clonic seizure | Bi-H | TLE | Bi-ANT | 3 | 3 | 0.0% | 69 |
| 11 | 45 | 17 | unknown | Focal impaired awareness sensory seizure | Bi-H | TLE | Bi-ANT | 6 | 3 | 55.6% | 59 |
| 12 | 29 | 16 | traumatic brain injury | Focal impaired awareness emotional seizure | T-PLUS | TLE | Bi-ANT | 9 | 2 | 78.6% | 78 |
| 13 | 18 | 12 | unknown | Focal cognitive seizure to bilateral tonic-clonic seizure | F, P | Multifocal | Bi-ANT | 24 | 11 | 52.8% | 37 |
| 14 | 27 | 18 | unknown | Focal impaired awareness autonomic seizure | T-PLUS | TLE | Bi-ANT | 390 | 24 | 93.8% | 41 |
| 15 | 28 | 8 | unknown | Focal impaired awareness autonomic seizure | Bi-T | TLE | Bi-ANT | 12 | 7 | 38.9% | 27 |
| 16 | 27 | 10 | encephalitis | Focal autonomic seizure to bilateral tonic-clonic seizure | Bi-T | TLE | Bi-ANT | 15 | 10 | 31.8% | 52 |
| 17 | 26 | 3 | encephalitis | Focal sensory seizure to bilateral tonic-clonic seizure | Bi-T | TLE | Bi-ANT | 39 | 1 | 97.4% | 83 |
| 18 | 22 | 10 | unknown | Focal autonomic seizure to bilateral tonic-clonic seizure | Bi-T | TLE | Bi-ANT | 7 | 3 | 63.6% | 67 |
| 19 | 22 | 15 | hypothalamic hamartoma | Focal autonomic seizure to bilateral tonic-clonic seizure | F | ETLE | Bi-ANT | 2 | 1 | 60.0% | 65 |
| 20 | 24 | 16 | unknown | Focal sensory seizure to bilateral tonic-clonic seizure | Bi-T | TLE | Bi-ANT | 12 | 1 | 91.4% | 41 |
| 21 | 17 | 10 | encephalitis | Focal sensory seizure to bilateral tonic-clonic seizure | Bi-T | TLE | Bi-ANT | 16 | 3 | 81.3% | 47 |
| 22 | 26 | 7 | encephalitis | Focal autonomic seizure to bilateral tonic-clonic seizure | Bi-T | TLE | Bi-ANT | 4 | 1 | 83.3% | 60 |
| 23 | 20 | 17 | unknown | Focal impaired awareness cognitive seizure | F, P,O | Multifocal | Bi-ANT | 30 | 24 | 19.1% | 38 |
| 24 | 26 | 4 | pesticide poisoning | Focal impaired awareness autonomic seizure | Bi-T | TLE | Bi-ANT | 4 | 1 | 63.6% | 53 |
| 25 | 14 | 5 | encephalitis | Focal impaired awareness cognitive seizure | Bi-H | TLE | Bi-ANT | 2 | 5 | -220.0% | 41 |
| 26 | 27 | 15 | right parietal ganglioglioma | Focal impaired awareness automatisms seizure | T-PLUS | TLE | Bi-ANT | 60 | 0 | 100.0% | 52 |
| 27 | 43 | 13 | traumatic brain injury | Focal autonomic seizure to bilateral tonic-clonic seizure | T-PLUS | TLE | Bi-ANT | 5 | 5 | 0.0% | 60 |
| 28 | 20 | 6 | encephalitis | Focal behavior arrest seizure to bilateral tonic-clonic seizure | Bi-H | TLE | Bi-ANT | 7 | 17 | -131.8% | 21 |
| 29 | 16 | 2 | encephalitis | Focal impaired awareness cognitive seizure | T-PLUS | TLE | Bi-ANT | 32 | 6 | 81.4% | 44 |
| 30 | 28 | 27 | sturge-weber syndrome | Focal autonomic seizure to bilateral tonic-clonic seizure | P,O | Multifocal | Bi-ANT | 4 | 0 | 91.7% | 46 |
| 31 | 25 | 17 | anoxia neonatorum | Focal automatisms seizure to bilateral tonic-clonic seizure | T-PLUS | TLE | Bi-ANT | 17 | 6 | 62.7% | 39 |
| 32 | 21 | 5 | unknown | Focal impaired awareness autonomic seizure | Bi-H | TLE | Bi-ANT | 1 | 1 | 0.0% | 44 |
| 33 | 21 | 14 | anoxia neonatorum | Focal impaired awareness automatisms seizure | Bi-H | TLE | Bi-ANT | 2 | 0 | 100.0% | 55 |
| 34 | 24 | 13 | unknown | Focal cognitive seizure to bilateral tonic-clonic seizure | Bi-H | TLE | Bi-ANT | 7 | 6 | 15.0% | 39 |
| 35 | 17 | 6 | meningitis | Focal behavior arrest seizure to bilateral tonic-clonic seizure | F | ETLE | Bi-ANT | 6 | 0 | 100.0% | 36 |
| 36 | 32 | 7 | unknown | Focal impaired awareness autonomic seizure | Bi-T | TLE | Bi-ANT | 10 | 2 | 76.7% | 36 |
| 37 | 33 | 9 | encephalitis | Focal impaired awareness clonic seizure | Bi-H | TLE | Bi-ANT | 25 | 9 | 64.0% | 26 |
| 38 | 30 | 19 | encephalitis | Focal cognitive seizure to bilateral tonic-clonic seizure | Bi-H | TLE | Bi-ANT | 4 | 1 | 75.0% | 24 |
| 39 | 30 | 11 | unknown | Focal tonic seizure to bilateral tonic-clonic seizure | Bi-H | TLE | Bi-ANT | 10 | 1 | 93.3% | 25 |
| 40 | 39 | 26 | unknown | Focal tonic seizure to bilateral tonic-clonic seizure | T-PLUS | TLE | Bi-ANT | 40 | 15 | 61.7% | 26 |
| 41 | 26 | 9 | encephalitis | Focal automatisms seizure to bilateral tonic-clonic seizure | Bi-T | TLE | Bi-ANT | 25 | 19 | 22.7% | 19 |
| 42 | 16 | 9 | encephalitis | Focal impaired awareness behavior arrest seizure | Bi-H | TLE | Bi-ANT | 3 | 3 | 0.0% | 20 |
| 43 | 25 | 6 | encephalitis | Focal tonic seizure to bilateral tonic-clonic seizure | Bi-H | TLE | Bi-ANT | 30 | 14 | 53.3% | 20 |
| 44 | 20 | 5 | encephalitis | Focal clonic seizure to bilateral tonic-clonic seizure | Bi-H | TLE | Bi-ANT | 18 | 17 | 9.1% | 18 |
| 45 | 36 | 13 | Gray Matter Heterotopia | Focal impaired awareness cognitive seizure | T-PLUS | TLE | Bi-ANT | 3 | 0 | 100.0% | 15 |
| 46 | 14 | 7 | Left rusmussen encephalitis | Focal aware clonic seizure | Frontoparietal | ETLE | L-STN | 300 | 2 | 99.0% | 108 |
| 47 | 33 | 26 | Bilateral schizencephaly | ①Focal aware tonic seizure; ②Focal to bilateral tonic–clonic seizure | Frontoparietal | ETLE | Bi-STN | ①90; ②GTCS 2/year | ①0.5; ②none | 99.0% | 36 |
| 48 | 14 | 10 | Left schizencephaly with FCD | Focal aware tonic seizure | Centrofrontal | ETLE | L-STN | 75 | 3 | 96.0% | 36 |
| 49 | 12 | 12 | Left schizencephaly | Focal aware tonic seizure | Centroparietal | ETLE | L-STN | 10 | none | 100.0% | 26 |
| 50 | 15 | 14 | Unknown | Focal aware tonic seizure；Focal to bilateral tonic–clonic seizure | Frontoparietal | ETLE | Bi-STN | ①44； ②4 | ①none; ②7 | -75.0% | 15 |
| 51 | 18 | 11 | Left precentral FCD | Focal aware tonic seizure | Centrofrontal | ETLE | L-STN | 6 | 1 | 83.3% | 12 |
| 52 | 15 | 13 | viral encephalitis | Focal impaired awareness tonic seizure | Centrofrontal | ETLE | Bi-STN | 300 | 90 | 71.0% | 26 |
| 53 | 19 | 17 | Unknown | Focal impaired awareness myoclonic seizure | Frontoparietal | ETLE | B-STN | 443 | 203 | 54.2% | 26 |
| 54 | 13 | 0.7 | Right rusmussen encephalitis | Focal aware clonic seizure | Frontoparietal | ETLE | R-STN | 120 | none | 100.0% | 25 |
| 55 | 27 | 19 | Bilateral schizencephaly | Focal to bilateral tonic–clonic seizure | Centroparietal | ETLE | B-STN | ①10; ②1 | none | 100.0% | 22 |
| 56 | 13 | 5 | Right precentral FCD | Focal aware tonic seizure | Centrofrontal | ETLE | Bi-STN | 30 | 7 | 76.7% | 18 |
| 57 | 36 | 17 | Left Frontal-parietal polymicrogyria | Focal to bilateral tonic–clonic seizure | Frontoparietal | ETLE | L-STN | 2-3 | 4 | -25.0% | 20 |
| 58 | 31 | 7 | Left Frontal FCD | Focal aware clonic seizure； Focal to bilateral tonic–clonic seizure | Centrofrontal | ETLE | L-STN | ①300; ②6 | ①180;②2 | 66.0% | 17 |
| 59 | 12 | 5 | Left paracentral lobule FCD | Focal to bilateral tonic–clonic seizure | Centrofrontal | ETLE | L-STN | 1 | none | 100.0% | 15 |
| 60 | 23 | 15 | Left side double cortex syndrome | Focal impaired awareness motor onset seizure | Centrofrontal | ETLE | L-STN | 3 | none | 100.0% | 12 |
| 61 | 18 | 18 | Bilateral perilateral fissure schizencephaly | Focal impaired awareness automatism seizure | Frontoparietal | ETLE | Bi-STN | 30 | 17 | 43.0% | 12 |
| 62 | 12 | 12 | unknown | atypical absence; tonic seizure; bilateral tonic-clonic seizure | unknown | unknown | Bi-CMN | 60 | 31 | 51.6% | 76 |
| 63 | 20 | 15 | unknown | atypical absence; tonic seizure | unknown | unknown | Bi-CMN | 113 | 90 | 79.6% | 43 |
| 64 | 16 | 7 | unknown | atypical absence; tonic seizure; bilateral tonic-clonic seizure | unknown | unknown | Bi-CMN | 83 | 66 | 79.5% | 12 |
| 65 | 23 | 20 | unknown | Focal cognitive seizure to bilateral tonic-clonic seizure | Bi-O | O | Bi-PN | 76 | 53 | 69.7% | 13 |

T, temporal; T-PLUS, temporal plus; F, frontal; C, central area; P, parietal; O, occipital; EZ, epileptogenic zone; FCD, focal cortical dysplasia; TLE, temporal lobe epilepsy; ETLE, extratemporal lobe epilepsy; L, left; R, right; Bi, bilateral; y, year; m, month.
